# Serum Uric Acid Concentrations and Risk of Adverse Outcomes in Patients With COVID-19

**DOI:** 10.3389/fendo.2021.633767

**Published:** 2021-05-06

**Authors:** Bo Chen, Chenyang Lu, Hong-Qiu Gu, Yang Li, Guqin Zhang, Jonathan Lio, Xiongyan Luo, Lingshu Zhang, Yidan Hu, Xiaomeng Lan, Zerong Chen, Qibing Xie, Huaqin Pan

**Affiliations:** ^1^ Department of Rheumatology and Immunology, West China Hospital, Sichuan University, Chengdu, China; ^2^ China National Clinical Research Center for Neurological Diseases, Beijing Tiantan Hospital, Capital Medical University, Beijing, China; ^3^ National Center for Healthcare Quality Management in Neurological Diseases, Beijing Tiantan Hospital, Capital Medical University, Beijing, China; ^4^ Department of Endocrinology, West China Hospital, Sichuan University, Chengdu, China; ^5^ Department of Respiratory and Critical Care Medicine, Zhongnan Hospital of Wuhan University, Wuhan, China; ^6^ Internal Medicine, University of Chicago, Chicago, IL, United States; ^7^ West China School of Public Health and West China Fourth Hospital, Sichuan University, Chengdu, China; ^8^ Department of Critical Care Medicine, Zhongnan Hospital of Wuhan University, Wuhan, China; ^9^ Department of Critical Care Medicine, Leishenshan Hospital, Wuhan, China; ^10^ Clinical Research Center of Hubei Critical Care Medicine, Wuhan, China

**Keywords:** COVID-19, uric acid, relationship, U-shape, adverse outcome

## Abstract

**Background:**

Although hyperuricemia frequently associates with respiratory diseases, patients with severe coronavirus disease 2019 (COVID-19) and severe acute respiratory syndrome (SARS) can show marked hypouricemia. Previous studies on the association of serum uric acid with risk of adverse outcomes related to COVID-19 have produced contradictory results. The precise relationship between admission serum uric acid and adverse outcomes in hospitalized patients is unknown.

**Methods:**

Data of patients affected by laboratory-confirmed COVID-19 and admitted to Leishenshan Hospital were retrospectively analyzed. The primary outcome was composite and comprised events, such as intensive care unit (ICU) admission, mechanical ventilation, or mortality. Logistic regression analysis was performed to explore the association between serum concentrations of uric acid and the composite outcome, as well as each of its components. To determine the association between serum uric acid and in-hospital adverse outcomes, serum uric acid was also categorized by restricted cubic spline, and the 95% confidence interval (CI) was used to estimate odds ratios (OR).

**Results:**

The study cohort included 1854 patients (mean age, 58 years; 52% women). The overall mean ± SD of serum levels of uric acid was 308 ± 96 µmol/L. Among them, 95 patients were admitted to ICU, 75 patients received mechanical ventilation, and 38 died. In total, 114 patients reached composite end-points (have either ICU admission, mechanical ventilation or death) during hospitalization. Compared with a reference group with estimated baseline serum uric acid of 279-422 µmol/L, serum uric acid values ≥ 423 µmol/L were associated with an increased risk of composite outcome (OR, 2.60; 95% CI, 1.07- 6.29) and mechanical ventilation (OR, 3.01; 95% CI, 1.06- 8.51). Serum uric acid ≤ 278 µmol/L was associated with an increased risk of the composite outcome (OR, 2.07; 95% CI, 1.18- 3.65), ICU admission (OR, 2.18; 95% CI, 1.17- 4.05]), and mechanical ventilation (OR, 2.13; 95% CI, 1.06- 4.28), as assessed by multivariate analysis.

**Conclusions:**

This study shows that the association between admission serum uric acid and composite outcome of COVID-19 patients was U-shaped. In particular, we found that compared with baseline serum uric acid levels of 279-422 µmol/L, values ≥ 423 µmol/L were associated with an increased risk of composite outcome and mechanical ventilation, whereas levels ≤ 278 µmol/L associated with increased risk of composite outcome, ICU admission and mechanical ventilation.

## Introduction

In December 2019, a cluster of patients with pneumonia, which was later identified as COVID-19, were identified in Wuhan. Thereafter, COVID-19 rapidly spread around the world (1), and, in November 25, 2020, the World Health Organization reported a total of 46,166,182 confirmed cases globally, with an average mortality being of 2.4% (2).

Patients with COVID-19 present with a variety of signs and symptoms as well as different prognoses, including recovery, admission to the intensive care unit (ICU), the need for mechanical ventilation, and death. Mild cases manifest fever and cough, whereas critical cases may manifest acute respiratory distress syndrome (ARDS), sepsis, or septic shock. Early detection of patients who are likely to develop critical disease is fundamental to identifying high-risk patients and allocating limited resources.

A high incidence of renal abnormalities and gastrointestinal symptoms has been reported in patients with COVID-19, and kidney diseases are frequently associated with mortality in these patients (3–6). The kidneys and gut are both targets of SARS-CoV-2 and the primary sites of uric acid excretion. Therefore, it is likely that infection with SARS-CoV-2 could affect regulation of uric acid metabolism and levels in the serum. Indeed, studies have shown that serum uric acid concentrations were markedly lower in patients with severe COVID-19 disease (7–9), which may be caused by decreased net renal tubular reabsorption of uric acid due to inflammation. In SARS-CoV-2-affected patients, hypouricemia was also found to be strongly associated with a poor prognosis (10). However, hyperuricemia is known to be associated with hypoxia and systemic inflammation in respiratory diseases (11). Angiotensin converting enzyme 2 (ACE2), the receptor for the entry of SARS-CoV-2, is strongly expressed in the kidney (12, 13), and SARS-CoV-2 can be detected in COVID-19 patients’ urine (14, 15). COVID-19-associated nephritis, which manifests as leukocyturia, albuminuria, and hematuria, is considered an early indicator of disease severity (16). Furthermore, a single-cell analysis showed enriched expression of ACE2 in all subtypes of proximal tubular cells of the kidney (13), which are the most important regulators of serum urate (17). A recent study also observed that uric acid significantly increases in children with severe COVID-19 compared with non-severe children on admission (18).

To date, no evidence on the precise association between serum concentrations of uric acid in COVID-19 patients on admission and in-hospital adverse outcomes exists. In this study, we investigated in detail the relationship between admission serum uric acid and the adverse outcomes in hospitalized patients.

## Methods

### Patients

This retrospective cohort study included 1854 adult patients (≥18 years old) admitted to Leishenshan Hospital, a hospital specifically built for COVID-19 treatment during disease outbreak, between February 16 and April 14, 2020, when the COVID-19 epidemic occurred in Wuhan (China). The diagnosis of COVID-19 was confirmed according to the WHO interim guidance. SARS-CoV-2 positivity was diagnosed by real-time reverse-transcriptase polymerase-chain-reaction (RT-PCR) assay conducted on nasal and pharyngeal swab specimens. Patients with incomplete or missing serum uric acid values within 24 hours after admission were excluded (n = 14). Patients with chronic kidney disease, gout, chronic liver disease with severe liver dysfunction, or malignancies (n = 50) were also excluded from the study. The flowchart of participants’ progress through the study is shown in **Supplementary Figure S1**.

Clinical data of all COVID-19 patients were collected. Due to the outbreak of the COVID-19 epidemic, written informed consent could not be collected from patients. We only made use of the deidentified retrospective data in this study. In addition, our study has been approved by the Ethics Committee of the Zhongnan Hospital of Wuhan University.

### Baseline Measurements and Definition

Relevant patients’ characteristics were recorded from electronic medical records, including age, sex, and comorbidities. Clinical and laboratory data were obtained within the first 24 hours of admission. They included vital signs, long-term use of medications, mode of respiratory support, complete blood count, coagulation profile, serum uric acid, creatinine, electrolytes, renal and liver function, lactate dehydrogenase, and D-dimer concentrations. Outcome data were also collected from electronic medical records. The Leishenshan hospital was closed on April 14, enabling complete extraction of outcome data. The primary outcome was a composite outcome of three different components, such as mortality, occurrence of mechanical ventilation and admission to the ICU. The following variables were associated with serum uric acid levels: age, sex, hypertension, diabetes, chronic lung disease, lymphocyte and platelet counts, aspartate aminotransferase, total bilirubin, albumin, creatine, C-reactive protein and D-dimer. An overnight fasting blood sample was collected to measure serum uric acid, aspartate aminotransferase, total bilirubin, albumin and creatinine.

Fever was defined as stated history or presence of axillary temperature of at least ≥37.5°C. The diagnosis of hypertension was based on a systolic blood pressure ≥140 mm Hg and/or diastolic blood pressure ≥90 mm, or by previous diagnosis of hypertension. Hypoproteinemia was defined as a serum albumin below 25 g/L.

### Outcomes

Complete outcome data were collected from electronic medical records. The primary outcome was a composite outcome defined as mortality or occurrence of mechanical ventilation or admission to the ICU. In our study, all participants were hospitalized patients with laboratory-confirmed COVID-19 and had a definite outcome (dead or discharged).

### Statistical Analysis

Participants were classified into three groups according to admission serum uric acid concentrations. The cut-off values of serum uric acid concentrations were based on the results of logistic regression with restricted cubic spline analysis. Continuous variables were reported as means ± standard deviation (SD) and categorical variables as frequencies expressed as percentages based on data distribution. Baseline characteristics among groups were compared using the χ^2^ test for categorical variables and analysis of variance or Kruskal–Wallis tests where appropriate.

Odds ratios for the association between plasma uric acid and composite outcome, ICU admission, mechanical ventilation, and mortality were estimated by modeling serum uric acid as a categorical variable using multivariable logistic regression. The covariates for poor outcome of COVID-19 were selected based on previous studies (19, 20), and were adjusted in multivariable logistic regression analyses. Serum uric acid concentrations were excluded at the 1% and 99% points. Three models were used: 1) age and sex were adjusted in multivariable models (Model 1); 2) comorbidities (hypertension, diabetes, and chronic lung disease) were added to the multivariable models (Model 2) laboratory tests (lymphocyte and platelet counts, aspartate aminotransferase, total bilirubin, albumin, creatine, C-reactive protein and D-dimer) were included in the multivariable models (Model 3).

Possible nonlinear relationships between serum uric acid and the composite outcome, ICU admission, mechanical ventilation, and death were examined with restricted cubic splines (21). Based on initial analysis of relationships between serum uric acid and primary outcome measurement, which revealed a nonlinear association, serum uric acid was analyzed as a continuous variable, fitting a restricted cubic spline function with three knots (located at the 5th, 50th, and 95th percentiles). Considering the restricted cubic spline analysis for serum uric acid and the composite outcome, 279–422 μmol/L was selected as the reference category. This range was associated with the lowest risk of all events, as multivariable cubic spline plots revealed significance thresholds at 279 μmol/L and 422 μmol/L.

R software (version 3.6.2; http://www.R-project.org), EmpowerStats and the IBM SPSS 25.0 software (IBM Corp., Armonk, NY, USA) were used for the statistical analyses. *P* < 0.05 was considered statistically significant in all analyses.

## Results

### Patient Characteristics

A total of 1854 participants (mean age, 58 years; 52% women) were included in this analysis (**Supplementary Figure S1**).

The mean age of patients was 58.1 ± 14.7 years and 48% were male. The mean SBP was 131.4 ± 15.7 mmHg, and 310 (16.7%) patients reported dyspnea. 478 (25.8%) patients had a history of hypertension, 222 (12%) had diabetes, 47 (2.5%) had chronic lung diseases, and 164 (8.8%) cardiovascular diseases. The overall mean serum uric acid level was 308.4 ± 95.9 μmol/L. Participants with higher serum uric acid concentrations were more likely younger, male, and showed higher SBP, and higher creatinine levels, as well as lower D-dimer. On the other hand, participants with lower uric acid concentrations were older, female, and showed lower SBP and creatine levels, as well as higher D-dimer levels (**Table 1**).

**Table 1:** Baseline patient characteristics by serum uric acid concentrations.

Characteristics	Overall (N = 1854)	Admission Serum Uric Acid Level, μmol/L			
		≤199 μmol/L (n = 200)	200-399 μmol/L (n = 1360)	≥400 μmol/L (n = 294)	*P*-value
**Baseline and demographic**					
Age	58.1 (14.7)	61.6 (14.9)	58.5 (14.2)	53.8 (15.9)	<0.001
Male	890 (48.0)	67 (33.5)	587 (43.2)	236 (80.3)	<0.001
SBP, mmHg	131.4 (15.7)	129.7 (15.4)	131.3 (15.8)	132.8 (15.4)	0.035
DBP, mmHg	81.1 (11.1)	78.0 (10.9)	81.1 (10.9)	83.2 (11.5)	<0.001
**Comorbidities**					
Hypertension	478 (25.8)	43 (21.5)	367 (27.0)	68 (23.1)	0.134
Diabetes	222 (12.0)	31 (15.5)	164 (12.1)	27 (9.2)	0.103
Chronic lung disease	47 (2.5)	6 (3.0)	33 (2.4)	8 (2.7)	0.869
Cardiovascular disease	164 (8.8)	24 (12.0)	108 (7.9)	32 (10.9)	0.068
**Lab test**					
Platelet, 10^9/L	238.51 ± 80.52	237.47 ± 83.11	235.07 ± 71.04	240.41 ± 80.48	0.604
Lymphocyte, 10^9/L					<0.001
<1	282 (14.9)	72 (36.0)	177 (13.0)	28 (9.5)	
≥1	1577 (85.1)	128 (64.0)	1183 (87.0)	266 (90.5)	
CRP, mg/L					0.004
<30	1736 (93.6)	722 (91.5)	212 (94.2)	802 (95.5)	
≥30	118 (6.4)	67 (8.5)	13 (5.8)	38 (4.5)	
AST, U/L					0.005
<40	1688 (91.0)	707 (89.6)	197 (87.6)	784 (93.3)	
≥40	166 (9.0)	82 (10.4)	28 (12.4)	56 (6.7)	
Total bilirubin, μmol/L	10.37 ± 5.34	10.35 ± 5.47	11.27 ± 6.34	10.15 ± 4.89	0.020
Albumin, g/L	37.11 ± 4.07	36.15 ± 4.07	38.86 ± 3.99	37.54 ± 3.85	<0.001
Creatinine, μmol/L					<0.001
<64.3	927 (50.0)	149 (74.5)	732 (53.8)	46 (15.6)	
≥64.3	927 (50.0)	51 (25.5)	628 (46.2)	248 (84.4)	
D-dimer, mg/L					<0.001
<1	1432 (77.2)	122 (61.0)	1064 (78.2)	246 (83.7)	
≥1	422 (22.8)	78 (39.0)	296 (21.8)	48 (16.3)	

Data are shown as mean (with SD) or n (%). P values comparing across three ranges of serum uric acid concentrations using χ^2^ tests for categorical variables and analysis of variance and Kruskal–Wallis tests for normally and nonnormally distributed continuous variables, respectively. SBP, systolic blood pressure; DBP, diastolic blood pressure. AST, aspartate aminotransferase; CRP, C-reactive protein.

### Association Between Serum Uric Acid and In-Hospital Outcomes

**Figure 1:**
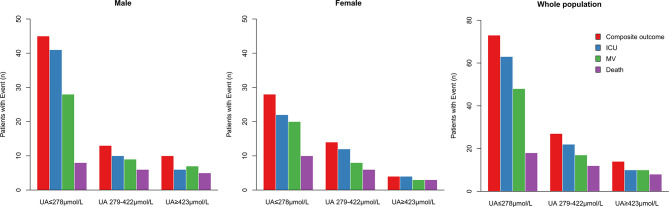
Distribution of males, females and whole population of COVID-19 patients reaching the composite outcome, ICU admission, mechanical ventilation, or death relatively to uric acid concentrations. Distribution of patients’ outcomes in the three ranges of serum uric acid concentrations from low to high (UA ≤ 278 μmol/L, UA 279–422 μmol/L, ≥ 423 μmol/L). ICU, intensive care unit; MV, mechanical ventilation.

During hospitalization, 95 subjects (5.1%) were admitted to ICU, 75 (4.0%) received mechanical ventilation, 38 (2.1%) undergone death, and 114 (6.1%) participants had composite outcome consisting of ICU admission, mechanical ventilation and death. Among the patients with uric acid ≤278 μmol/L (*n* = 789), 73 (9.3%) received composite outcome, 63 (8%) had ICU admission, 48 (6%) with mechanical ventilation, and 18 (2.3%) died. In contrast, among the patients with uric acid ≥423 μmol/L (*n* = 225), 14 (6.2%) subjects had composite outcome, 10 (4.4%) had ICU admission, 10 (4.4%) received mechanical ventilation, and 8 (3.6%) died (**Table 2**, **Figure 1**).

**Table 2:** Association Between Outcomes and Serum Uric Acid by Categories at Admission.

Outcome	No.of patients	Event (%)	Model 1		Model 2		Model 3	
			OR (95% CI)	*P* value	OR (95% CI)	P value	OR (95% CI)	*P* value
**Composite Outcome**		114 (6.1)						
UA ≤ 278 μmol/L	789	73	3.05 (1.92- 4.86)	<0.001	3.02 (1.89- 4.82)	<0.001	2.07 (1.18- 3.65)	0.011
UA 279- 422 μmol/L	840	27	1 [Reference]		1 [Reference]		1 [Reference]	
UA ≥423 μmol/L	225	14	2.14 (1.07- 4.25)	0.031	2.10 (1.05- 4.21)	0.036	2.60 (1.07- 6.29)	0.035
**ICU admission**		95 (5.1)						
UA ≤ 278 μmol/L	789	63	3.19 (1.92- 5.30)	<0.001	3.13 (1.88- 5.21)	<0.001	2.18 (1.17- 4.05)	0.014
UA 279- 422 μmol/L	840	22	1 [Reference]		1 [Reference]		1 [Reference]	
UA ≥423 μmol/L	225	10	1.81 (0.83- 3.98)	0.0172	1.74 (0.79- 3.86)	0.267	1.75 (0.63- 4.85)	0.281
**Mechanical Ventilation**		75 (4.0)						
UA ≤ 278 μmol/L	789	48	3.04 (1.71- 5.38)	<0.001	3.01 (1.70- 5.36)	<0.001	2.13 (1.06- 4.28)	0.033
UA 279- 422 μmol/L	840	17	1 [Reference]		1 [Reference]		1 [Reference]	
UA ≥423 μmol/L	225	10	2.41 (1.06- 5.46)	0.036	2.42 (1.06- 5.50)	0.048	3.01 (1.06- 8.51)	0.038
**Death**		38 (2.1)						
UA ≤ 278 μmol/L	789	18	1.40 (0.66- 2.97)	0.383	1.40 (0.66- 2.97)	0.388	0.92 (0.33- 2.60)	0.876
UA 279- 422 μmol/L	840	12	1 [Reference]		1 [Reference]		1 [Reference]	
UA ≥423 μmol/L	225	8	3.01 (1.16- 7.81)	0.023	3.05 (1.16- 7.98)	0.026	3.94 (0.99- 15.8)	0.052

Model 1: Adjusted for age, sex. Model 2: Model 1 plus hypertension, diabetes, and chronic lung disease. Model 3: Model 2 plus adjustment for lymphocyte, platelet, aspartate aminotransferase, total bilirubin, albumin, Creatinine, C-reactive protein and D-dimer. ICU, intensive care unit; MV, mechanical ventilation; UA, uric acid.

**Figure 2:**
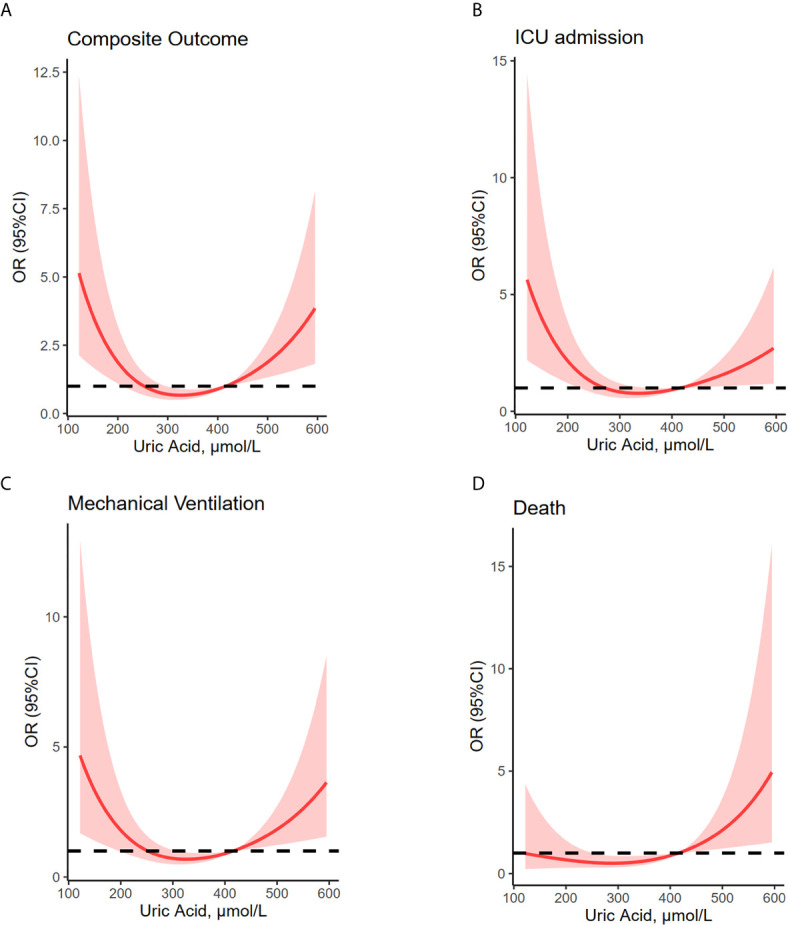
U-shaped association between uric acid concentrations and composite outcome, ICU admission, mechanical ventilation and death. Restricted multivariable cubic spline plots show U-shaped associations between admission serum uric acid and composite outcome **(A)**, ICU admission **(B)**, mechanical ventilation **(C)**, and death **(D)**. OR were adjusted for age, sex, hypertension, diabetes, lung disease, creatine, lymphocyte and platelet counts, aspartate aminotransferase, total bilirubin, albumin, creatine, C-reactive protein and D-dimer values. Median uric acid concentrations (279–422 μmol/L) have been considered as reference. Shaded areas are 95% CI derived from restricted cubic spline regressions with three spaced knots at the 5th, 50th, and 95th percentiles. The dashed line indicates OR equal to 1. ICU, intensive care unit; MV, mechanical ventilation.

Restricted multivariable cubic spline analysis was performed to analyze the relationship between serum uric acid and adverse outcomes of COVID-19 (**Table 2**, **Figure 2**).

### Composite Outcome

Associations between serum uric acid and composite outcome were U-shaped. The composite outcome occurred in 114 (6.1%) subjects. Statistical models were fully adjusted for several factors, including age, sex, hypertension, diabetes, chronic lung disease, lymphocyte and platelet counts, aspartate aminotransferase, total bilirubin, albumin, creatine, C-reactive protein and D-dimer levels. These models showed that compared with baseline serum uric acid of 279 to 422 µmol/L (*n* = 840; 3.2% with the composite outcome), both higher and lower serum uric acid levels (OR, 2.60; 95% CI, 1.07- 6.29 for ≥423 µmol/L and OR, 2.07; 95% CI, 1.18- 3.65 for ≤278 µmol/L, respectively) were associated with an increased risk of composite outcome consisting of ICU admission, mechanical ventilation, and death.

### ICU Admission

A total of 95 (5.1%) COVID-19 patients were admitted to ICU. Compared with baseline serum uric acid concentrations (279-422 µmol/L) shown by the 2.6% of subjects admitted to ICU, lower uric acid levels were significantly associated with increased risk of ICU admission (OR, 2.18; 95% CI, 1.17- 4.05 for ≤278 µmol/L).

### Mechanical Ventilation

A total of 75 patients received mechanical ventilation. Among them the 2.0% showed serum uric acid baseline concentrations. Levels of serum uric acid higher and lower than 279-422 µmol/L were associated with an increased risk of receiving mechanical ventilation (OR, 3.01; 95% CI, 1.06- 8.51 for ≥ 423 µmol/L and OR, 2.13; 95% CI, 1.06- 4.28 for ≤278 µmol/L, respectively).

### Death

Overall, 38 patients of the study cohort died. Among them, the 1.4% had baseline uric acid levels. Levels of serum uric acid higher than 279-422 µmol/L was associated with increased risk of death (OR, 3.94; 95% CI, 0.99- 15.8 for ≥ 423 µmol/L).

## Discussion

In this study, we report that serum concentrations of uric acid and adverse clinical outcomes of COVID-19 patients are U-shaped associated, thus suggesting that uric acid values on admission can be an independent predictor of prognosis. In fact, higher baseline serum uric acid levels were associated with an increased risk of composite outcome and mechanical ventilation. Similarly, lower baseline serum uric acid levels were associated with increased risk of composite outcome, ICU admission, and mechanical ventilation. Threshold effect analysis showed that serum uric acid levels between 279 and 422 µmol/L can be considered generally safe with respect to poor outcomes. To our knowledge, this is the first study describing serum uric acid, a widely available and low-cost diagnostic biomarker, as a predictor of adverse outcomes in COVID-19 patients. Among others used, this parameter might be useful for the identification of high-risk COVID-19 patients who could benefit from intensive management.

Hyperuricemia is frequently found in patients affected by chronic obstructive pulmonary disease (COPD) or other respiratory diseases (22–24). High level of uric acid can also be detected in children with severe COVID-19 on admission (18). However, patients with severe coronavirus disease 2019 (COVID-19) and severe acute respiratory syndrome (SARS) have been shown to develop marked hypouricemia (7–9). Thus, the association between uric acid and outcomes of COVID-19 is still under debate. The present study found that both high and low uric acid were associated with increased risk of adverse clinical outcomes. A smooth curve was fitted to show the U-shaped relationship between serum uric acid and composite outcome, as well as each of its components.

Angiotensin converting enzyme 2 (ACE2), the receptor for the entry of SARS-CoV-2, is strongly expressed in the kidney (12, 13), and SARS-CoV-2 can be detected in COVID-19 patients’ urine (14, 15). COVID-19-associated nephritis, which manifests as leukocyturia, albuminuria, and hematuria, is considered an early indicator of disease severity (16). Furthermore, a single-cell analysis showed enriched expression of ACE2 in all subtypes of proximal tubular cells of the kidney (13), which are the most important regulators of serum urate (17). SARS-CoV-2 infection is likely to cause uric acid dysregulation, resulting in abnormal serum uric acid concentrations. Moreover, increased uric acid excretion has been observed in patients with respiratory failure (25). It has been shown that proinflammatory cytokines can influence uric acid excretion or serum uric acid level (10, 26). For example, serum IL-8 level positively correlated with fraction excretion of uric acid while negatively correlated with serum uric acid level in SARS patients (10); whereas IL-6 level during gouty attach was correlated with serum uric acid change (26). Although little is known about the effects of cytokines on uric acid transporting, it is speculated that some cytokines modulate activities of specific channels (such as calcium channels and sodium channels) or transporters (such as SGLT2) by various mechanisms (27–29), and thus may affect functions of urate transporters including URAT1 and GLUT9 (30, 31). Therefore, the cytokine storm syndrome initiated by SARS-CoV-2 infection may play a role in the uric acid disruption in COVID-19 patients.

Uric acid is an important antioxidant, scavenging reactive oxygen species and free radicals. A prospective randomized controlled clinical trial showed that a nucleotide-supplemented diet significantly reduces complications and shortens the hospital stay of ICU patients (32), suggesting serum uric acid as a possible surrogate of antioxidant capacity. Moreover, uric acid crystals can be sensed by Clec12A, a regulator of type-I interferon responses, which is a pivotal defensive mechanism against viral infection (33). Uric acid strongly enhances T-cell immune responses to viruses (34), and low uric acid concentrations increase the risk of infection with viruses, such as Epstein–Barr virus (35). However, few studies on associations between serum uric acid concentrations and infections have been previously reported. Hypouricemia is a poor prognostic indicator in patients with intra-abdominal sepsis (36) or radiating pneumonitis (37). Another study showed that hypouricemic patients with SARS had poorer outcomes, especially in terms of respiratory failure (10). Consistent with this, we observed that COVID-19 patients with hypouricemia had higher risks of admission to ICU and mechanical ventilation. It remains unclear whether poor outcomes in hypouricemic patients with COVID-19 are in part due to a shortage of antioxidants.

Nevertheless, high uric acid concentrations can have direct pathophysiological effects, including increased oxidative stress, inflammation, endothelial dysfunction, activation of the renin–angiotensin–aldosterone system, and insulin resistance (17). Hyperuricemia has been found to be associated with various diseases, including coronary heart disease (38), hypertension (39), kidney failure (40), and chronic obstructive pulmonary disease exacerbations (22). It is likely that we have identified a phenomenon that reflects complex interactions between uric acid and other risk factors.

The present study had some limitations. First, the observation design did not permit to establish causality between serum uric acid concentrations and mortalities, ICU admission, and requirement for mechanical ventilation. In addition, residual confounding may persist, even after multivariable adjustment. We only analyzed serum concentrations of uric acid on admission and did not have access to follow-up measurements over time, and some vital variables such as fraction excretion of uric acid were unavailable.

Nevertheless, our findings could be useful to predict whether ICU or mechanical ventilation will be needed and, thus, to optimize patient allocation for special therapies and initiation of strategies to save lives.

In conclusion, the present study has shown a U-shaped association between serum uric acid levels and composite outcome, thus suggesting a prognostic role of serum uric acid for COVID-19 patients.

Our findings suggest that more attention should be paid to serum uric acid levels in the evaluation of COVID-19 disease status. This study also suggests a possible role for serum uric acid in the identification of COVID-19 patients at high risk of adverse clinical outcomes that need early intensive management. More studies are needed to establish a safe range values of serum uric acid in patients with COVID-19, which is urgent for patients who are taking uric acid-lowing drugs, and to clarify the molecular basis of this relationship.

## Data Availability Statement

The original contributions presented in the study are included in the article/**Supplementary Material**. Further inquiries can be directed to the corresponding authors.

## Ethics Statement

The studies involving human participants were reviewed and approved by the research ethics board of Zhongnan Hospital of Wuhan University, Wuhan, China. Written informed consent from the patients was not required to participate in this study in accordance with the national legislation and the institutional requirements.

## Author Contributions

HP, GZ, XYL, LZ, ZL, YH, XML, and ZC collected the data. BC and HG performed the statistical analysis. HP and QX supervised the project. BC, CL, and YLwrote the manuscript. HG, JL, and QX revised the manuscript. All authors contributed to the article and approved the submitted version.

## Funding

Scientific and Technological Project of COVID-19, West China Hospital, Sichuan University (HX-2019-nCoV-032) and National Natural Science Foundation of China (Grant no. 81700493).

## Conflict of Interest

The authors declare that the research was conducted in the absence of any commercial or financial relationships that could be construed as a potential conflict of interest.

## Supplementary Material

Supplementary Figure 1 Flowchart summarizing the selection of study participants.
