# Supplementary figures and images for: Serum Uric Acid Concentrations and Risk of Adverse Outcomes in Patients With COVID-19
Source: Front Endocrinol (Lausanne). 2021 May 6;12:633767. doi: 10.3389/fendo.2021.633767 (PMC8134697; doi:10.3389/fendo.2021.633767)

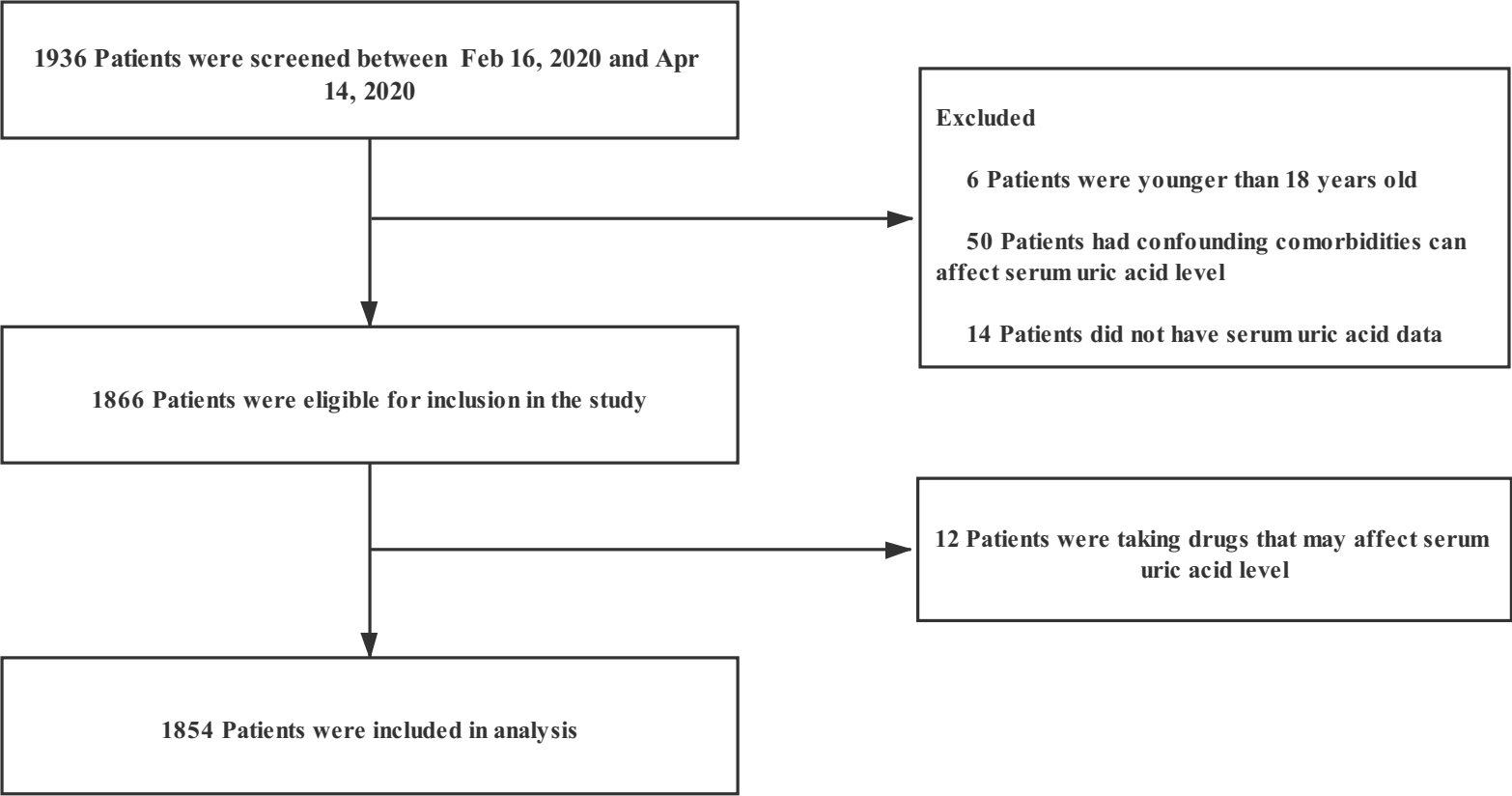

Supplement: Supplementary Figure 1 — Flowchart summarizing the selection of study participants. [file DataSheet_1.pdf]
